# Non-random distribution of *Plasmodium* Species infections and associated clinical features in children in the lake Victoria region, Kenya, 2012–2018

**DOI:** 10.1186/s41182-024-00622-3

**Published:** 2024-08-05

**Authors:** Protus Omondi, Brian Musyoka, Takatsugu Okai, James Kongere, Wataru Kagaya, Chim W. Chan, Mtakai Ngara, Bernard N. Kanoi, Yasutoshi Kido, Jesse Gitaka, Akira Kaneko

**Affiliations:** 1 Department of Virology, Graduate School of Medicine, Osaka Metropolitan University, Osaka, Japan; 2 Department of Parasitology/ Osaka International Research Center for Infectious Diseases, Osaka Metropolitan University, Osaka, Japan; 3 Department of Eco-Epidemiology, Institute of Tropical Medicine, Nagasaki University, Nagasaki, Japan; 4 Department of Clinical Medicine, Mount Kenya University, Thika, Kenya; 5 Island Malaria Group, Department of Microbiology, Tumor and Cell Biology, Karolinska Institutet, Stockholm, Sweden

**Keywords:** Mixed *Plasmodium* infection, *Plasmodium falciparum*, *Plasmodium malariae*, *Plasmodium ovale*, Kenya

## Abstract

**Background:**

While *Plasmodium falciparum* (Pf**)** stands out as the most lethal malaria parasite species in humans, the impact of other species should not be dismissed. Moreover, there is a notable lack of understanding of mixed-species infections and their clinical implications.

**Methods:**

We conducted eight school-based cross-sectional malariometric surveys in the Lake Victoria region of western Kenya between January–February 2012 and September–October 2018. In each survey, a minimum of 100 children aged 3 to 15 years were randomly chosen from a school in Ungoye village on the mainland and as well as from each school selected in every catchment area on Mfangano island. *Plasmodium* infection was determined by microscopy and nested polymerase chain reaction (PCR). The multiple-kind lottery (MKL) model calculated the expected distribution of *Plasmodium* infections in the population and compared it to observed values using a chi-squared test (χ^2^).

**Results:**

The *Plasmodium* prevalence was 25.9% (2521/9724) by microscopy and 51.1% (4969/9724) by PCR. Among all infections detected by PCR, Pf, *P. malariae* (Pm)*,* and *P. ovale* (Po) mono-infections were 58.6%, 3.1%, and 1.8%, respectively. Pf/Pm, Pf/Po, Pm/Po*,* and Pf/Pm/Po co-infections were 23.5%, 4.3%, 0.1%, and 8.6%, respectively. MKL modelling revealed non-random distributions, with frequencies of Pf/Pm and Pf/Pm/Po co-infections being significantly higher than expected (χ^2^ = 3385.60, *p* < 0.001). Pf co-infections with Pm and Po were associated with a decreased risk of fever (aOR 0.64, 95% CI 0.46–0.83; *p* = 0.01) and increased risks of splenomegaly (aOR 12.79, 95% CI 9.69–16.9; *p* < 0.001) and anaemia (aOR 2.57, 95% CI 2.09–3.15; *p* < 0.001), compared to single-species infections.

**Conclusion:**

This study sheds light on the potential interaction between Pf and Pm and/or Po. Given the clinical significance of mixed-species infections, improved diagnostics, and case management of Pm and Po are urgently needed.

**Supplementary Information:**

The online version contains supplementary material available at 10.1186/s41182-024-00622-3.

## Introduction

Malaria remains a significant global public health concern, especially in sub-Saharan Africa. In 2022, there were estimated 249 million malaria cases in 85 endemic countries, resulting in 608,000 deaths according to the World Health Organization (WHO) [1]. Alarmingly, children under the age of five accounted for 76% of these malaria-related fatalities [1]. Efforts to control malaria in sub-Saharan Africa have primarily focused on *Plasmodium falciparum* (Pf), the most lethal human *Plasmodium* species. However, *P. malariae* (Pm) and *P. ovale* (Po) are endemic in many parts of sub-Saharan Africa and mixed-species infections are common, with nearly all possible combinations observed within human populations [2–4].

Interactions between co-occurring *Plasmodium* species can influence infection dynamics, species prevalence, and distribution due to resource competition, varying levels of virulence, and the host's immune response to each species [5–8]. The consequences of co-infection on infection virulence have been well documented in rodent malaria, as controlled experiments with human malaria are not feasible. Tang et al. [8] found that co-infections of *P. yoelii* with either *P. vinckei* or *P. chabaudi* significantly increased infection virulence, resulting in 100% mortality in mixed species infections. In contrast, single infections of *P. yoelii* and *P. vinckei* caused no mortality, and *P. chabaudi* alone caused 40% mortality. Previous studies have linked these interspecies interactions to either increased or reduced likelihood of severe malaria development in children, depending on the parasite species involved. For instance, a prospective cohort study from Papua New Guinea (PNG) found that *P. vivax* and mixed infections were associated with severe malaria in children [9]. In contrast, coinfections with Pm were found to reduce the severity of fevers in children in West Africa [10]. Mixed-species infections remain a persistent challenge to malaria elimination efforts due to their variability in case management and transmission prevention. Natural interactions among *Plasmodium* species in humans also appear to differ by geography, which can result from differences in climate and genetic characteristics in host and vector populations [11, 12].

**Fig. 1:**
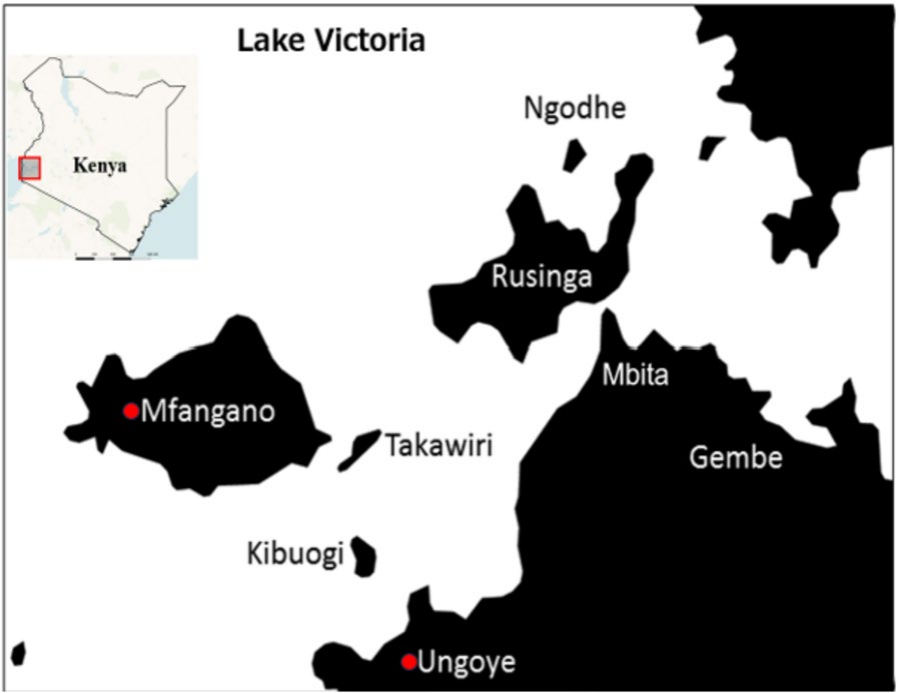
The Lake Victoria basin of Homabay County in western Kenya where the malaria cross-sectional surveys were conducted

With a surface area of 69,485 km^2^ and an altitude of 1,135 m above sea level, Lake Victoria is the world's second-largest freshwater lake. In Homa Bay County, Kenya, the Lake Victoria basin encompasses two populous islands, Rusinga and Mfangano, as well as several smaller inhabited islands (Fig. 1). Due to the year-round availability of suitable breeding sites, these islands and lakeside villages have a high density of *Anopheles* vectors [13]. Our earlier study in the region revealed that most PCR-detected *Plasmodium* infections were asymptomatic (91.8%) and submicroscopic (50.3%). We reported a prevalence of 13.2% for non-*falciparum* (*P. malariae* and *P. ovale*) infections and 11.3% for mixed *Plasmodium* infections by PCR. Additionally, the lakeshore community of Ungoye and Mfangano island showed a higher prevalence of non-*falciparum* and mixed *Plasmodium* infections than smaller islands [14]. Another study conducted among health facilities in six different regions of Kenya found that non-*falciparum* and mixed infections comprised 27.5% and 25.8%, respectively, of symptomatic cases [15]; however, they did not reflect the community prevalence. Apart from the difference in a wide range of ecological factors, such as climate and vector-human interactions, these differences suggest distinct geographical niches for specific species. Differential species burden patterns observed across malaria-endemic areas highlight the need for improved diagnostic tools and a better understanding of the epidemiology of mixed *Plasmodium* and *non-falciparum* malaria and their clinical implications [12, 16].

To date, mixed *Plasmodium* infections have frequently been overlooked or underestimated when microscopy is used. While molecular diagnostic methods have revealed that non-*falciparum* single and mixed infections are more common throughout sub-Saharan Africa and other malaria-endemic regions of the world, their epidemiology remains far less studied [17–20]. The clinical implications of these non-*falciparum* species, which may differ from those of Pf, are poorly understood within endemic human populations. The main concern of missed mixed infections is that inadequate treatment or disease management could result in debilitating infections and their associated economic and social consequences after Pf is eliminated.

In this study, we conducted repeated cross-sectional surveys between 2012 and 2018 in the Lake Victoria region of Western Kenya. Our goal was to determine the distribution of mixed malaria infections using molecular diagnostic methods and identify clinically relevant features of these infections. We employed a multiple-kind lottery model (MKL) to investigate whether the occurrence of different *Plasmodium* species is independent of species-to-species interactions and to evaluate changes in the distribution of various parasite species combinations in the study population for six years. We further analysed the effect of single-species and mixed infections with Pf, Pm, and Po on the clinical features of malaria recorded on the day of enrolment using logistic regression models.

## Methods

### Ethical approval

The study was conducted in accordance with the Declaration of Helsinki and was approved by the Kenyatta National Hospital/University of Nairobi-Ethics and Research Committee in Kenya (No. P7/1/2012), the Mount Kenya University Independent Ethics and Research Committee (MKU-IERC) and the Ethics Committee at Osaka Metropolitan University (approval number 3206). Local interpreters explained the purposes and procedures of the study to the survey subjects, and written informed consent was obtained from each adult participant during study registration. For children, written informed consent was obtained from their parents or legal guardians.

### Study sites

Ungoye (19.3 km^2^) is a village on Lake Victoria's shore in Homa Bay County, Kenya. According to the 2019 Kenya Population and Housing Census [21], it had a population of about 5000. Mfangano Island (66 km^2^) is approximately 12 km northwest of Ungoye and has a population of 25000 (Fig. 1). This region typically experiences a short rainy season between November and December and a long rainy season between March and June. Peak malaria transmission occurs 4–8 weeks after the rainy season and is often associated with up to eight-fold increases in malaria vector abundance [22]. The relative abundance of malaria vector species and suitable larval habitats has been linked with the high and heterogeneous malaria prevalence between neighbouring Islands and the mainland in Lake Victoria [14].

### Study design

Eight cross-sectional school-based malariometric surveys were conducted between January–February 2012 and September–October 2018. In Ungoye the same primary school was sampled throughout the study period. On Mfangano, catchment areas were defined by geographic features (coastal lowland vs. interior mountains) and proximity to population centres and major villages, and corresponded approximately to the east coast, highland, and west coast as described by Idris et al. [14]. At least one public primary school within each catchment area, totalling four to six schools per survey were sampled to adequately cover the entirety of the island. We obtained lists of school children from selected schools and systematically selected a minimum of 100 children at random to participate in the study. For six years, the surveys were conducted about two months after the rainy seasons, specifically in October-December and February–March, to coincide with the periods with the highest malaria transmission.

### Field methods and laboratory procedures

Before sample collection, appropriate collection and processing protocols were designed to ensure samples were collected and identified correctly. The sex and age of each participant were recorded at enrolment. Axillary temperature was determined using a digital thermometer (Terumo, Tokyo, Japan). Participants aged 12 years and under were examined for clinically palpable spleen according to Hackett's method [23] by author AK.

Finger-prick blood samples were collected from each participant for the determination of haemoglobin level by HemoCue Hb 201 + analyser (HemoCue, Ängelholm, Sweden) and *Plasmodium* infections by rapid diagnostic test (RDT), light microscopy, and PCR. *P. falciparum* infections were diagnosed on-site using the Paracheck *Pf* RDT (Orchid Biomedical System, Goa, India), and RDT-positive participants were given artemether-lumefantrine treatments with instructions according to the Ministry of Health's case management guidelines for uncomplicated malaria. Thick and thin blood smears were made on a glass slide. The thin blood smear was fixed with methanol, and both smears were stained with 3% Giemsa solution for 30 min. Samples were examined by two independent experienced microscopists and considered negative if no parasites were observed after examination of 100 fields under 1000X magnification. A third experienced microscopist, who was blinded to the results from the initial two examinations, resolved any discrepancies. Two blood spots (70 μl each) were blotted on Whatman 31ET Chr filter paper (Cytiva Life Sciences, Tokyo, Japan) using heparinised microhaematocrit capillary tubes (Thermo Fisher Scientific, USA). Blood spots were allowed to dry at ambient temperature, packed in individual zippered plastic bags containing silica gel desiccant and stored at -20 °C. The QIAamp Blood Mini Kit (QIAGEN, Germantown, USA) was used to extract DNA from a quartered blood spot (17.5 μl) according to the manufacturer's instructions. A nested PCR procedure described by Isozumi et al. [24], which can differentiate the four primary human malaria species except *P. knowlesi*, was used to detect *Plasmodium* infection. Negative controls and positive controls (purified *Plasmodium* DNA from BEI Resources, Manassas, Virginia, USA) were included in both primary and secondary PCRs.

### Variable definitions

A *Plasmodium* infection was defined by positive PCR of one or more *Plasmodium* species. Samples that were positive by microscopy but negative by PCR were treated as negative in any analysis. Fever was defined as an axillary temperature ≥ 37.5 °C, and infections without fever were considered asymptomatic. The age groups were defined as below 5 years, 5–10 years, and 11–15 years, as defined before in our setting [14]. Non-anaemia, mild and moderate anaemia, and severe anaemia for children in each age category were defined according to the World Health Organization standard [25]. Severe malarial anaemia was defined as Hb ≤ 5 g/dL in the presence of any malaria parasite by microscopy and/or PCR [26]. Splenomegaly was defined using Hackett's spleen size classification in a score of 0–5, reflecting the degree of splenic enlargement [23].

### Data management and statistical methods

All analyses were done using Stata/SE 16.1 (StataCorp, Texas, USA) and R version 4.2.2 (The R Foundation for Statistical Computing, Vienna, Austria). Descriptive statistics were calculated for each study site yearly. Means were compared between study sites using Student's t-test or Mann–Whitney rank test, while proportions were compared using Pearson's Chi-square tests or Fisher exact tests. The average enlarged spleen (AES) index was calculated as the sum of the number of children in each spleen size class multiplied by the class number (0–5), divided by the total number of palpable spleen. The MKL model [27] was used to tabulate the species' expected numbers and all the combinations in each population (more detail about the model provided in the supplementary materials). The Chi-square tests for heterogeneity were used to compare observed with expected numbers of infections. Univariable and multivariable mixed effects logistic regression models generated adjusted odds ratios (aOR) and 95% confidence intervals (CI) adjusting for age, sex, and survey year to assess the association between single and mixed *Plasmodium* infection status, fever, splenomegaly (clinically palpable spleen), and anaemia, with school set as the random effect. The analysis was performed using the glmer function in the lme4 R package. A multinomial logistic regression model was used to evaluate the relative risk ratio of Pf mono-infections jointly, Pf mixed-species infections, and non-*falciparum* infections over time while adjusting for the above-specified covariates.

## Results

### Study participants

We analysed data from 9724 school children aged between 3 and 15 years, with 6286 children from Mfangano and 3438 from Ungoye, throughout the study period (Table 1). Among these participants, slightly more than half were females (50.9%), and the average age was 8.2 years. Most participants belonged to age categories of either 5–10 or 11–15-year (80.4%; 95% Confidence Interval CI 78.5–82.4).

**Table 1:** Participants characteristics by study site

Sample characteristics	Total (*n* = 9724)	Mfangano (*n* = 6286)	Ungoye (*n* = 3438)
Year			
2012	2424	1443	981
2013	1013	440	573
2014	1543	674	869
2015	1532	1398	134
2017	1723	1090	633
2018	1489	1241	248
Mean age in years (SD)	8.2 (3.7)	8.4 (3.7)	7.7 (3.8)
Age group *n* (%) in years			
< 5	1903 (19.6)	1095(17.4)	808 (23.5)
5–10	4715 (48.5)	3093 (49.2)	1622 (47.2)
11–15	3106 (33.2)	2098 (33.4)	1008 (29.3)
Sex = Male *n* (%)	4770 (49.1)	3082 (49.0)	1688 (49.1)
Haemoglobin^a^ levels *n* (%)			
Normal	6984 (72.7)	4682 (75.0)	2302 (68.6)
Mild anaemia	1159 (12.1)	683 (10.9)	476 (14.2)
Moderate anaemia	1333 (13.9)	793 (12.7)	540 (16.1)
Severe anaemia	126 (1.3)	88 (1.4)	38 (1.0)
Auxiliary temperature^b^ = fever prevalence n/N (%)	1313/9679 (13.6)	804/6263 (12.8)	509/3416 (14.9)
Splenomegaly^c^ prevalence n/N (%)	3188/7004 (45.5)	1645/4400 (37.4)	1543/2604 (59.2)

^a^Anaemia defined according to WHO standard (n = 122 missing)

^b^Fever was defined as an axillary temperature ≥ 37.5 °C (n = 45 missing)

^c^Splenomegaly was screened only on children ≤ 12 years (n = 7004)

SD standard deviation, % percentage

### *Plasmodium* species-specific prevalence and clinical outcomes

The overall prevalence of *Plasmodium* infections in Ungoye was significantly (*p* < 0.001) higher than that in Mfangano, and this difference was observed throughout the study period by both PCR and microscopy (Supplementary Fig. 1).

**Fig. 2:**
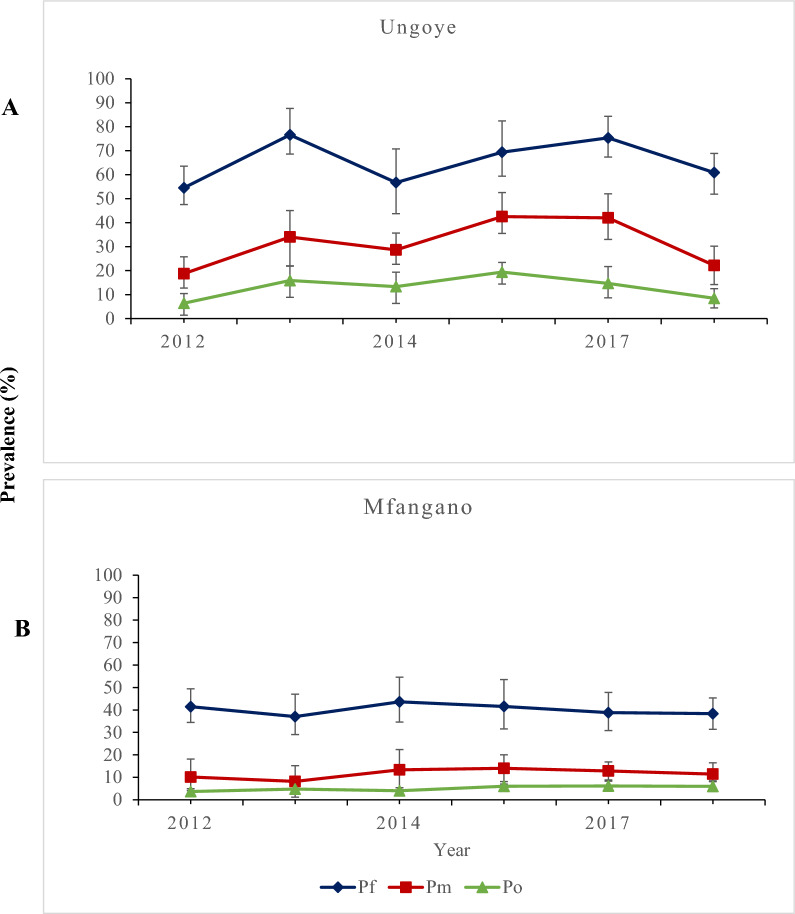
Yearly (2012–2018) *Plasmodium* species-specific prevalence detected by polymerase chain reaction. **A** Ungoye. **B** Mfangano island. Error bars represent 95% confidence intervals. No surveys were conducted in 2016. *Pf Plasmodium falciparum*, *Pm Plasmodium malariae*, *Po Plasmodium ovale*

In both Ungoye and Mfangano, the species-specific prevalence of Pf, Pm, and Po remained consistent between 2012 and 2018 (Fig. 2). Species-specific prevalence of all three species as determined by PCR and microscopy was dependent on age (*p* < 0.001), with the lowest overall prevalence observed in children under the age of 5 (Supplementary Fig. 2). By PCR, the 11–15 age group (n = 3106) had the highest Pf- (53.1%; 95% CI 50.8–56.1) and Pm-specific (18.9%; 95% CI 16.2–19.8) prevalence, while the 5–10 age group (n = 4715) had the highest Po-specific prevalence (8.4%; 95% CI 6.5–9.6,).

Of all participants, 1313 (13.6%; 95% CI 12.8–14.3) were febrile, and 2618 (27.3%; 95% CI 25.7–28.8) had a haemoglobin measurement below 11 g/dl. Of all the children with anaemia, 1159 (12.1%; 95% CI 11.4–12.7), 1333 (13.2%; 95% CI 13.2–14.6), and 126 (1.1%; 95% CI 1.1–1.6) had mild, moderate, and severe anaemia, respectively, based on the WHO standard. Among those screened for splenomegaly (aged 3–12 years), 3188 (45.5%; 95% CI 44.3–46.7) were found to have an enlarged spleen, with an AES index of 1.73. The prevalence of enlarged spleen was significantly (*p* < 0.001) higher in Ungoye (59.2%; 95% CI 57.3–61.1, AES = 1.78) compared to Mfangano (37.3%; 95% CI 35.9–38.8, AES = 1.70) (Table 1).

### *Plasmodium* species infections

**Fig. 3:**
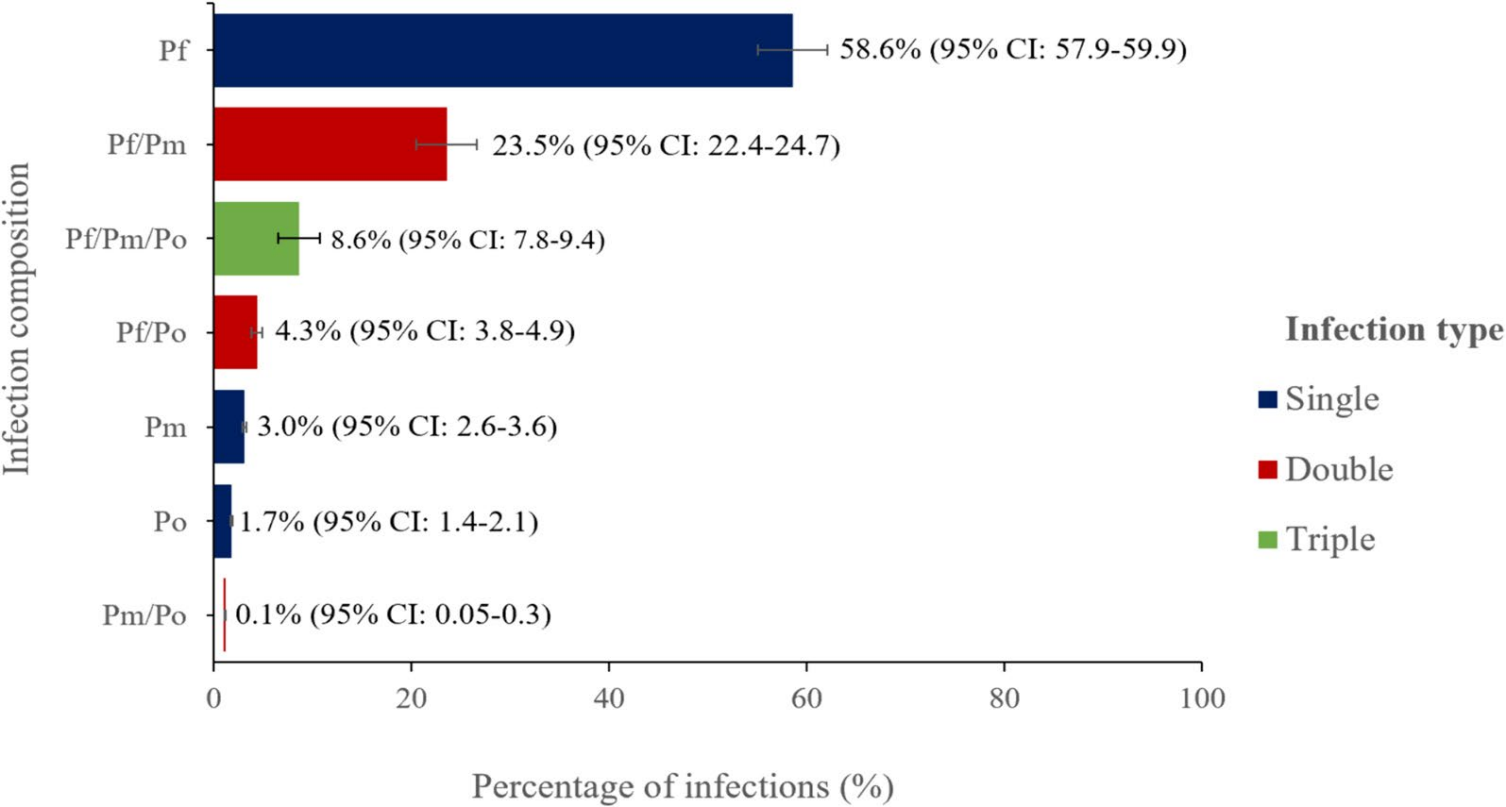
Proportion of *Plasmodium* mono and mixed-species infections among the positive cases by polymerase chain reaction (n = 4969). The error bars represent a 95% confidence interval (CI). Pf *Plasmodium falciparum*, Pm *Plasmodium malariae*, Po *Plasmodium ovale*

Out of the total 9724 children included in the study, PCR detected 4969 *Plasmodium* infections (51.1%; 95% CI 50.1–52.1). The majority, 2910 (58.6%; 95% CI 57.2–59.9), were identified as Pf mono-infections, 153 (3.0%; 95% CI 2.6–3.6) as Pm mono-infections, and 87 (1.7%; 95% CI 1.4–2.1) as Po mono-infections. Furthermore, there were 1170 cases (23.5%; 95% CI 22.4–24.7) of Pf/Pm, 216 (4.3%; 95% CI 3.8–4.9) Pf/Po, 6 (0.1%; 95% CI 0.05–0.3) Pm/Po, and 427 (8.6%; 95% CI 7.8–9.4) Pf/Pm/Po mixed infections (Fig. 3). A similar trend in proportion was observed when data was analysed across the sites. However, the proportion of mixed infections involving Pf (Pf/Pm and Pf/Pm/Po) was significantly higher in Ungoye compared to Mfangano island (Pf/Pm: 29.9%; 95% CI 28.0–31.8 vs 18.2%; 95% CI 16.8–19.7, *p* = 0.002, and Pf/Pm/Po: 12.6%; 95% CI 11.3–14.0 vs 5.3%; 95% CI 4.5–6.2, *p* < 0.001, Supplementary Fig. 3).

### Distribution of mixed-species infections

The frequencies of *Plasmodium* assemblages deviated significantly from the random pattern predicted by the MKL model for both PCR (χ2 = 3385.60; df = 7; *p* < 0.001) and microscopy (χ2 = 459.73; df = 7; *p* < 0.001) (Table 2). We observed higher-than-expected numbers of Pf/Pm and Pf/Pm/Po mixed infections, while the numbers of Pf/Po and Pm/Po mixed infections and single-species (Pf, Pm and Po) infections were lower than expected (Table 2).

**Table 2:** Observed and expected frequencies of *Plasmodium* species infections by PCR and Microscopy

Species composition	PCR (*n* = 9724)			Microscopy (*n* = 9724)		
	Observed	Expected	Chi-square	Observed	Expected	Chi-square
Pf	2910	3577.18	124.43	2270	2399.08	6.94
Pm	153	834.75	556.79	48	165.15	83.10
Po	87	310.17	160.57	8	25.53	12.04
Pf/Pm	1170	788.34	184.77	168	56.05	223.59
Pf/Po	216	292.92	20.20	21	8.67	17.55
Pm/Po	6	68.35	56.88	1	0.60	0.27
Pf/Pm/Po	427	64.56	2034.95	5	0.20	113.67
Not infected	4755	3787.73	247.01	7203	7068.72	2.55
	Chi-square = (df = 7)3385.60; p < 0.001			Chi-square = (df = 7) 459.73; p < 0.001		

Chi-square values were calculated using heterogeneity tests (number of rows × number of columns) to compare observed versus expected values

*df* degrees of freedom, Pf *Plasmodium falciparum*, Pm *Plasmodium malariae*, Po *Plasmodium ovale*

Next, we analysed whether the distribution pattern of *Plasmodium* infections was influenced by age or study site. We categorised the children into three age groups: below 5 years (n = 1903), 5–10 years (n = 4715), and 11–15 years (n = 3106). By PCR, significant (all *p* < 0.001) deviations from a random distribution pattern were observed for all age groups (Table 3). By study site, significant deviations were observed in both Ungoye (χ2 = 1621.17; df = 7; *p* < 0.001) and Mfangano (χ2 = 1211.89; df = 7; *p* < 0.001) (Table 4).

**Table 3:** Observed and expected frequencies of *Plasmodium* species infections by age group-PCR analysis

Species composition	< 5 years (n = 1903)			5–10 years (n = 4715)			11–15 years (n = 3106)		
	Ob served	Expected	Chi-square	Ob served	Expected	Chi-square	Ob served	Expected	Chi-square
Pf	514	627.24	20.44	1376	1701.77	62.36	1020	1240.92	39.33
Pm	16	135.69	105.58	84	418.73	267.58	53	271.45	175.79
Po	25	75.66	33.92	43	165.69	90.84	19	71.75	38.78
Pf/Pm	147	94.86	28.65	562	395.42	70.18	461	307.61	76.48
Pf/Po	36	52.90	5.40	111	156.46	13.21	69	81.31	1.86
Pm/Po	1	11.44	9.53	2	38.50	34.60	3	17.79	12.29
Pf/Pm/Po	86	8.00	760.51	241	36.36	1151.96	100	20.16	316.29
Not infected	1078	897.20	36.43	2296	1802.09	135.37	1381	1095.02	74.69
	Chi-square = (df = 7)1000.47; p < 0.001			Chi-square = (df = 7) 1826.11; p < 0.001			Chi-square = (df = 7) 735.52; p < 0.001		

*df* degrees of freedom, Pf *Plasmodium falciparum*, Pm *Plasmodium malariae*, Po *Plasmodium ovale*

**Table 4:** Observed and expected frequencies of *Plasmodium* species infections by study site-PCR analysis

Species composition	Mfangano (*n* = 6286)			Ungoye (*n* = 3438)		
	Observed	Expected	Chi-square	Observed	Expected	Chi-square
Pf	1780	2116.76	53.58	1130	1363.19	39.89
Pm	109	424.33	234.33	44	322.15	240.16
Po	61	171.32	71.04	26	105.45	59.86
Pf/Pm	494	286.77	149.75	676	563.88	22.29
Pf/Po	118	115.78	0.04	98	184.58	40.61
Pm/Po	4	23.21	15.90	2	43.62	39.71
Pf/Pm/Po	143	15.69	1033.34	284	76.35	564.73
Not infected	3577	3132.14	63.18	1178	778.79	204.64
	Chi-square = (df = 7) 1621.17; p < 0.001			Chi-square = (df = 7) 1211.89; p < 0.001		

*df* degrees of freedom, Pf *Plasmodium falciparum*, Pm *Plasmodium malariae*, Po *Plasmodium ovale*

### Association with clinical measures of malaria

**Fig. 4:**
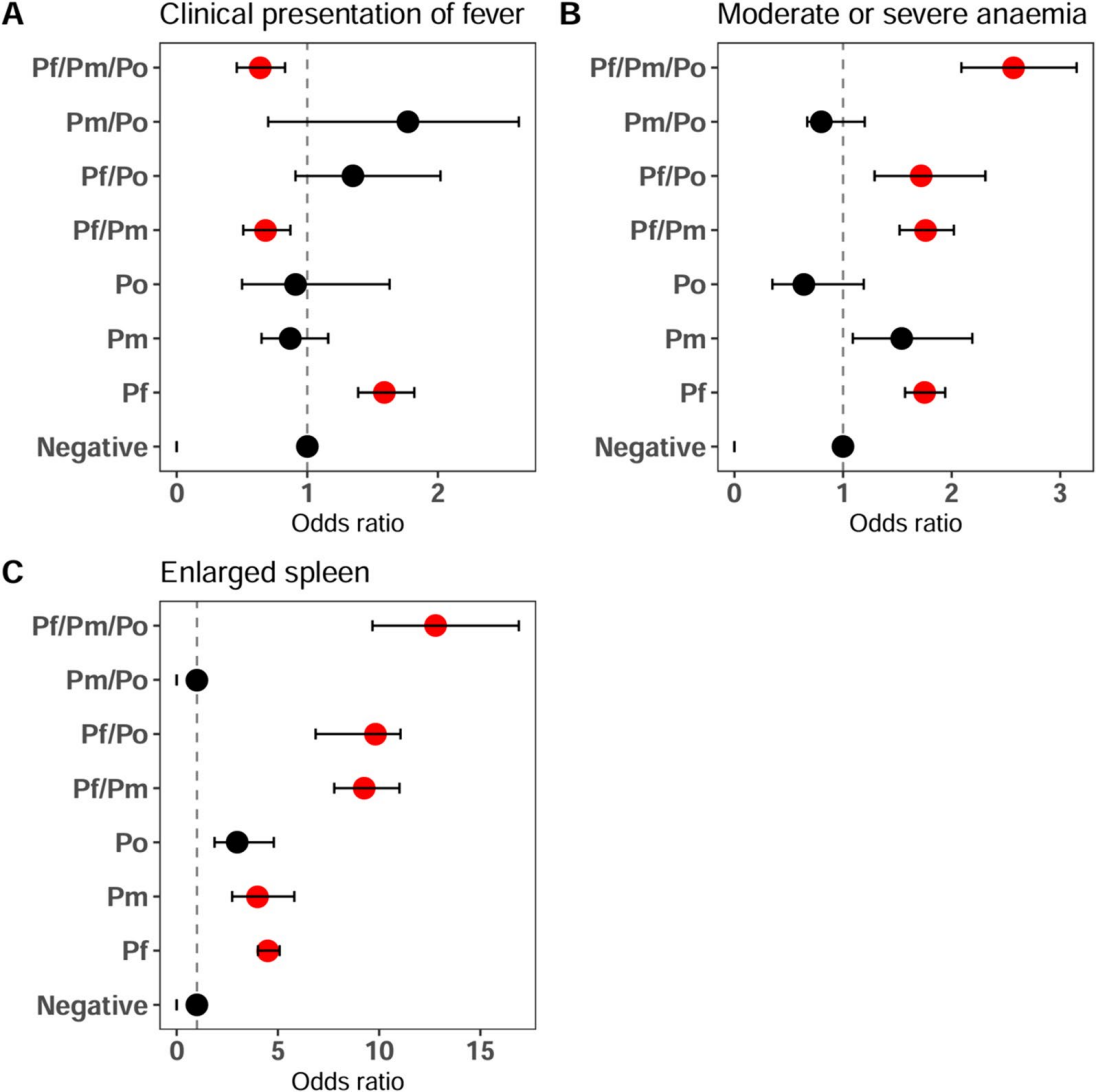
Clinical features association with Plasmodium single and mixed species infection. **A** Clinical fever at presentation defined as an axillary temperature ≥ 37.5 °C, **B** Moderate or severe malarial anaemia defined according to WHO standard, **C** Enlarged spleen according to Hackett's method. The odds ratios are adjusted for age, sex, school, and year of the survey. The error bars indicate the 95% confidence interval**.** *Pf Plasmodium falciparum*, *Pm Plasmodium malariae*, *Po Plasmodium ovale*. Significant odds ratios (p > 0.001), are indicated in red

After adjusting for age, sex, study site, and year of survey in logistic regression models, Pf/Pm (aOR = 0.68, 95% CI 0.51–0.87, *p* = 0.03) and Pf/Pm/Po (aOR = 0.64, 95% CI 0.46–0.83, *p* = 0.01) mixed infections were significantly associated with reduced risks of fever, while Pf mono-infections were significantly (aOR = 1.59, 95% CI (1.39–1.82, *p* < 0.01) associated with an increased risk of fever. Pf mono-infections and mixed-infections with Pm and/or Po were significantly associated with anaemia and palpable spleen when compared to infections caused by a single species, as shown in Fig. 4.

### Risk for mixed *Plasmodium* species infections

We investigated the risk factors associated with infections caused by single and multiple species of *Plasmodium* in children. Both Pf and Pf/Pm or Pf/Pm/Po showed significant association with age group, the use of insecticide-treated nets (ITNs), and the location of the study. Notably, the likelihood of contracting Pf mixed infections, specifically Pf/Pm and Pf/Pm/Po, was approximately four and six times higher, respectively (aOR = 4.15; 95% CI 3.63–4.74 and aOR = 6.03; 95% CI 4.88–7.45, *p* < 0.001) in children from Ungoye compared to those from Mfangano (Table 5).

**Table 5:** Risk Factors for single and mixed *Plasmodium* infections in children

Species composition	Variable	Category	cOR	95% CI	*P* value	aOR	95% CI	*P* value
Pf mono-infection	Age group (years)	< 5	1.00	…	…	1.00	…	…
		5–10	1.25	1.10–1.42	< 0.001	1.33	1.17–1.53	< 0.001
		11–15	1.54	1.35–1.77	< 0.001	1.69	1.46–1.95	< 0.001
	Study site	Mfangano						
		Ungoye	1.92	1.74–2.12	< 0.001	1.93	1.73–2.15	< 0.001
	Sleep under ITN last night	No	1.00	…	…	1.00	…	…
		Yes	0.86	0.76–0.95	0.006	0.90	0.81–1.01	0.077
Pm mono-infection	Age group (years)	< 5	1.00	…	…	1.00	…	…
		5–10	2.46	1.43–4.23	0.001	2.37	1.35–4.18	0.003
		11–15	2.58	1.46–4.55	0.001	2.60	1.44–4.70	0.002
	Study site	Mfangano	1.00	…	…	1.00	…	…
		Ungoye	1.22	0.85–1.75	0.263	1.38	0.94–2.01	0.100
	Sleep under ITN last night	No	1.00	…	…	1.00	…	…
		Yes	0.79	0.56–1.11	0.186	0.85	0.60–1.20	0.359
Po mono-infection	Age group (years)	< 5	1.00	…	…	1.00	…	…
		5–10	0.81	0.49–1.32	0.400	0.68	0.38–1.18	0.171
		11–15	0.59	0.32–1.08	0.089	0.49	0.24–0.99	0.047
	Study site	Mfangano	1.00	…	…	1.00	…	…
		Ungoye	1.29	0.81–2.06	0.276	1.60	0.95–2.69	0.076
	Sleep under ITN last night	No	1.00	…	…	1.00	…	…
		Yes	0.72	0.48–1.06	0.103	0.71	0.48–1.06	0.096
Pf/Pm	Age group (years)	< 5	1.00	…	…	1.00	…	…
		5–10	1.79	1.47–2.18	< 0.001	2.13	1.72–2.64	< 0.001
		11–15	2.44	2.00–2.99	< 0.001	2.96	2.38–3.69	< 0.001
	Study site	Mfangano	1.00	…	…	1.00	…	…
		Ungoye	4.15	3.63–4.74	< 0.001	4.71	4.07–5.46	< 0.001
	Sleep under ITN last night	No	1.00	…	…	1.00	…	…
		Yes	0.66	0.57–0.77	< 0.001	0.76	0.65–0.89	0.001
Pf/Po	Age group (years)	< 5	1.00	…	…	1.00	…	…
		5–10	1.45	0.98–2.12	0.058	1.60	1.05–2.41	0.026
		11–15	1.50	0.99–2.25	0.055	1.67	1.07–2.60	0.024
	Study site	Mfangano	1.00	…	…	1.00	…	…
		Ungoye	2.52	1.91–3.32	< 0.001	2.80	2.07–3.80	< 0.001
	Sleep under ITN last night	No	1.00	…	…	1.00	…	…
		Yes	0.65	0.47–0.90	0.009	0.68	0.49–0.94	0.021
Pm/Po	Age group (years)	< 5	1.00	…	…	1.00	…	…
		5–10	0.93	0.08–10.37	0.959	1.09	0.09–12.23	0.943
		11–15	2.34	0.24–22.54	0.461	1.86	0.17–20.7	0.614
	Study site	Mfangano	1.00	…	…	1.00	…	…
		Ungoye	1.51	0.27–8.30	0.630	1.96	0.32–12.01	0.466
	Sleep under ITN last night	No	1.00	…	…	1.00	…	…
		Yes	0.16	0.02–1.40	0.097	0.18	0.20–1.65	0.131
Pf/Pm/Po	Age group (years)	< 5	1.00	…	…	1.00	…	…
		5–10	1.31	1.02–1.70	0.036	1.57	1.19–2.07	0.001
		11–15	0.91	0.67–1.22	0.525	1.07	0.78–1.48	0.646
	Study site	Mfangano	1.00	…	…	1.00	…	…
		Ungoye	6.03	4.88–7.45	< 0.001	7.01	5.56–8.86	< 0.001
	Sleep under ITN last night	No	1.00	…	…	1.00	…	…
		Yes	0.69	0.55–0.88	0.003	0.77	0.60–0.98	0.036
Non-infected	Reference							

All odds ratios are adjusted for the above covariates and year of survey

Pf *Plasmodium falciparum*, Pm *Plasmodium malariae*, Po *Plasmodium ovale*, *cOR* crude odds ratio, *aOR* adjusted odds ratio

The multinomial logistic model was also used to estimate the relative risk ratio of all the single and mixed *Plasmodium* infections compared to being uninfected over time. The model-predicted adjusted probability and relative risk ratio of mixed infections involving Pf and Pm with and without Po (Pf/Pm and Pf/Pm/Po) and Po mono-infection increased significantly (*p* < 0.001), while the probability of Pf mono-infection decreased significantly (*p* < 0.001). (Supplementary Table 1, Supplementary Fig. 4).

## Discussion

Detailed analysis of results from a series of cross-sectional school surveys in the Lake Victoria basin of Kenya showed that *Plasmodium* species were non-randomly distributed among infections. Higher-than-expected numbers of Pf/Pm and Pf/Pm/Po co-infections were observed when the infections were diagnosed by microscopy or PCR, and when the population was stratified by age groups and geographical settings Interestingly, Pf/Pm and Pf/Pm/Po co-infections were associated with a reduced risk of fever but an increased risk of splenomegaly and anemia compared to single-species infections.

Our findings of non-random association of *Plasmodium* species are consistent with previous studies in sub-Saharan Africa [28–30] but contrast with those in PNG [31, 32]. These variations could be attributed to the different sets of sympatric species included [11]. Non-random distribution occurs when one parasite species either excludes another or when they are transmitted together, suggesting an effective interspecific interaction [27]. Malaria parasites are known to engage in mutual suppression in infected hosts, however, which species suppress the other is not well understood [11, 33]. The most straightforward explanations are through competition of nutrients and red blood cells (RBCs) [33, 34]. Whereas Pm and Po infect only mature and young (reticulocytes) RBCs respectively, Pf prefers young RBCs but is capable of invading all RBC age classes [35]. Hence, high Pf parasite density may reduce the availability of RBCs for invasion by other species [33]. McQueen and McKenzie [36] found that competition for RBCs generally increased *P. vivax* parasitemia in mixed-species malaria infections, although this effect varied based on the species' relative reproduction rates and the timing of inoculation.

Activation of nonspecific host-defence mechanisms could also restrict the proliferation of the accompanying parasite, especially during the rapid exponential growth phase [37]. Clinical studies have shown the existence of cross-species immunity in human malaria species [38, 39]. However as reported by Bruce et al. [7], when the majority population (in our case Pf which constitutes the majority of the infections in children) is cleared by species-specific immunity, total density will drop below the threshold that can trigger suppression (density-dependent regulation). In these circumstances, the minority population (Pm and Po) could expand because there is no competition [7]. This could explain why mixed *Plasmodium* infections (double infection of Pf and Pm*,* or triple infection of Pf, Pm and Po) are common in our study population, which primarily comprise asymptomatic and sub-microscopic infections. With respect to malaria control and elimination, our findings suggest that if species-specific vaccines or treatments successfully prevent or reduce Pf infections in highly endemic areas, the absence of constraints imposed by density-dependent regulation could allow non-*falciparum *species like Pm and Po to proliferate. Consequently, misdiagnosis and suboptimal drug treatment (e.g. no primaquine or tafenoquine radical treatment for Po) can allow the latent species to persist with serious clinical repercussions [33].

Additionally, the extent to which persistent parasitaemia in asymptomatic mixed species infections is a result of density-dependent regulation or immune response to numerous antigenic variants, along with its clinical implications, remains poorly understood [40]. Our findings indicate that children with mixed infections comprising double infections of Pf and Pm, or triple infections of Pf, Pm and Po had lower odds of having fever compared to those with single Pf infections at enrolment (Fig. 4A). Black et al. [10] suggested that Pm infections ameliorate the fevers of subsequent Pf superinfections in West African children due to constant exposure to parasites by frequent re-infection or long-term survival of parasites at low parasitaemia. Two previous studies conducted in Malawi and Tanzania similarly demonstrated that children with mixed *Plasmodium* infections had significantly reduced parasite density compared to children with single Pf but mixed infections were not correlated with fever reduction [41, 42].

However, mixed infections were also associated with unfavourable clinical features, double (Pf/Pm) and triple co-infections (Pf/Pm/Po) were more strongly associated with moderate or severe anaemia compared to children with single Pf infections (Fig. 4B). The contribution of persistent parasitaemia at sub-microscopic levels in mixed infections to the development of anaemia remains uncertain, and there is still controversy about the role of asymptomatic infections in infected children [43]. Nonetheless, it has been proposed that prolonged infections with the slowly replicating Pm and Po lead to continuous destruction of both parasitised and non-parasitised RBCs [44]. We also found an association between co-infections comprising Pf and Pm*,* both with and without Po and splenomegaly (Fig. 4C), which aligns with a previous study in Uganda [30]. There is conflicting evidence on spleen enlargement in children with mixed infections compared to those with single-species infections [45, 46], but our finding adds more evidence to the clinical relevance of infection with non-*falciparum* species within mixed-species infections.

We observed that the risk of Pf/Pm and Pf/Pm/Po mixed infections was significantly higher in children from Ungoye compared to those from Mfangano (Table 5). The high malaria prevalence in Ungoye has been linked to unpaved roads and extensive irrigation for farming, which create ample favourable *Anopheles* larval habitats [13]. Field studies have demonstrated that at least seven *Anopheles* species can carry more than one human *Plasmodium* species [47]. For instance, *An. gambiae* can carry all four major human *Plasmodium* species simultaneously [48]. Since female anopheline mosquitoes feed on human blood multiple times throughout their lives, areas with high malaria transmission could see both sequential and simultaneous transmission of different *Plasmodium* species and genotypes within species. Nonetheless, other non-entomological factors such as household structure features, occupation, and individual attractiveness to vector bites may influence the variation in mixed *Plasmodium* infections in our study area, but they are not fully understood.

The MKL model utilises prevalence data to provide a more realistic approximation of malaria infections in natural settings, as some hosts are infected with more than one parasite species simultaneously while others remain uninfected [27, 32]. However, it is important to acknowledge that this study focused exclusively on the prevalence of infection, not parasitaemia or parasite density. However, since PCR-based diagnosis is sufficiently sensitive to provide a comprehensive assessment of parasites involved in individual infections, it was possible to determine if there was evidence for cross-species interaction (non-random distribution) statistically. Further investigations on parasite density using quantitative molecular tests and other statistical models will be beneficial in providing a full assessment of the species-specific effect on parasite density. The cross-sectional nature of our sample collections also precluded us from examining how each *Plasmodium* species in mixed-species infections was acquired and whether one species may persist longer than others. Furthermore, the clinical effects of mixed infections could not be monitored longitudinally. School-based surveys are efficient and target the age groups with the highest *Plasmodium* prevalence in our study area [14], however, it remains unclear whether the pattern of excess Pf/Pm and Pf/Pm/Po infections and their association with negative clinical features are observed in adults who have more robust immunity against Pf. Another limitation of our study is that children from the same school in our study were sampled over time, which means some children might have participated in more than one surveys. Artemether-lumefantrine treatment cleared parasitaemia in RDT-positive cases during the school surveys. However, parasitaemia in sub-microscopic infections, which were detected only by PCR, is persistent with decreasing parasite density over an extended period [49]. Given the high entomological inoculation rates (EIRs) in our study area [50], this persistent parasitaemia is gradually dominated by new infections. In each school survey, we detected most probably new infections, even though some children were repeatedly surveyed. As mentioned above, the results of infections detected by microscopy and those by PCR were not different.

In conclusion, PCR of blood samples from a series of cross-sectional surveys in school children in a region characterized by high and heterogeneous prevalence of malaria [14] has demonstrated that mixed *Plasmodium* infections are remarkably common and non-randomly distributed. Given the clinical significance of mixed-species infections, improved diagnostics, and case management of Pm and Po are urgently needed.

### Supplementary Information

Additional file 1.

## Data Availability

The data used during the analysis is available upon reasonable request from the corresponding author.
