# Supplementary material for: Non-random distribution of Plasmodium Species infections and associated clinical features in children in the lake Victoria region, Kenya, 2012–2018
Source: Trop Med Health. 2024 Aug 5;52:52. doi: 10.1186/s41182-024-00622-3 (PMC11299388; doi:10.1186/s41182-024-00622-3)
Supplement: Supplementary file 1 — Additional file 1. [file 41182_2024_622_MOESM1_ESM.docx]

**Supplementary materials**

**Multiple Kind Lottery model**

The multiple-kind lottery (MKL) model was employed to calculate the expected numbers of parasite species infection assemblages in each population. This model assumes:

1. Acquisition of infection is an independent event.
2. One possible outcome of exposure is no acquisition of infection.

In this context:

- *p1*,*p2*, and *p3* represent the probabilities of infection by *P. falciparum*, *P. malariae*, and *P. ovale*, respectively.
- *q1*,*q2*, and *q3​* represent the probabilities of absence of infection by *P. falciparum, P. malariae*, and *P. ovale*, respectively.

The expected frequency of infection patterns are calculated as follows:

- Infection by *P. falciparum* only: *p1q2q3* ​
- Infection by *P. falciparum* and *P. malariae*: *p1p2q3*
- Infection by *P. falciparum* and *P. malariae and P. ovale*: *p1p2p3*
- No infection: *q1q2q3*
- Chi-square values were computed using heterogeneity tests to compare observed values with these expected frequencies.

The multiple-kind lottery model is a valuable tool for understanding the dynamics of multispecies malaria infections and their potential implications for public health. However, researchers should consider its limitations and complement its findings with data from other approaches to gain a more comprehensive understanding of malaria parasite dynamics.

Understanding how multiple species interact, this model can inform the development of effective public health strategies, such as vaccination and drug treatment approaches.

**A**

Prevalence (%)

**B**

**Supplementary Figure 1. Yearly malaria prevalence by polymerase chain reaction (PCR) and Microscopy.** A) Ungoye. B) Mfangano island. Error bars represent 95% confidence intervals. No surveys were conducted in 2016.

**A**

**Prevalence (%)**

**B**

**Supplemenatry Figure 2. Age-specific prevalence of *Plasmodium* spp. in Lake Victoria**. **A)** Age-specific prevalence by Polymerase chain reaction (PCR). **B)** Age-specific prevalence by Microscopy. The error bars represent a 95% confidence interval (CI). Abbreviations: Pf*, Plasmodium falciparum;* Pm*, Plasmodium malariae,* Po*, Plasmodium ovale,* PCR, Polymerase chain reaction. *Overall, represents joint *Plasmodium* spp. prevalence by PCR in A and by microscopy in B.


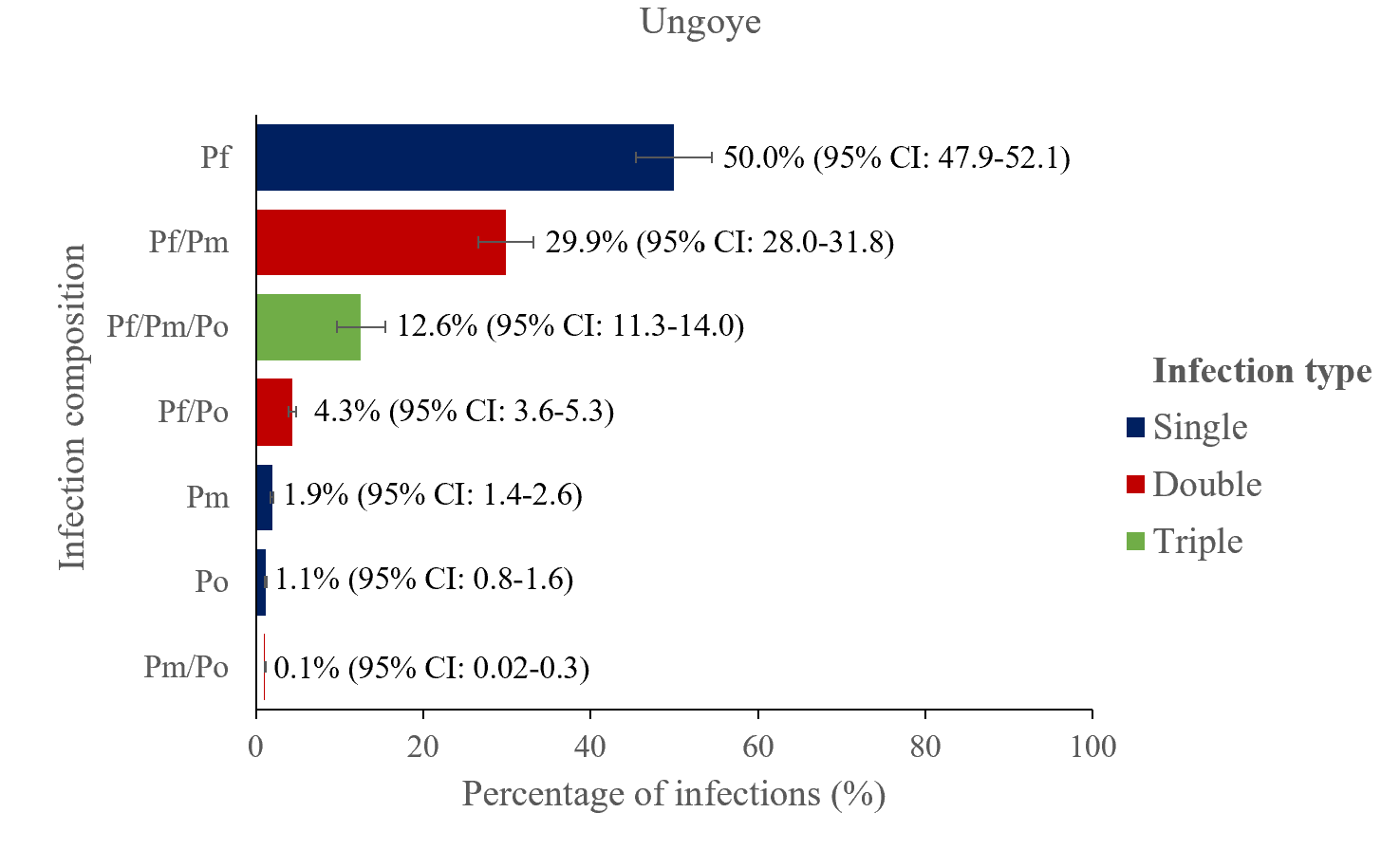

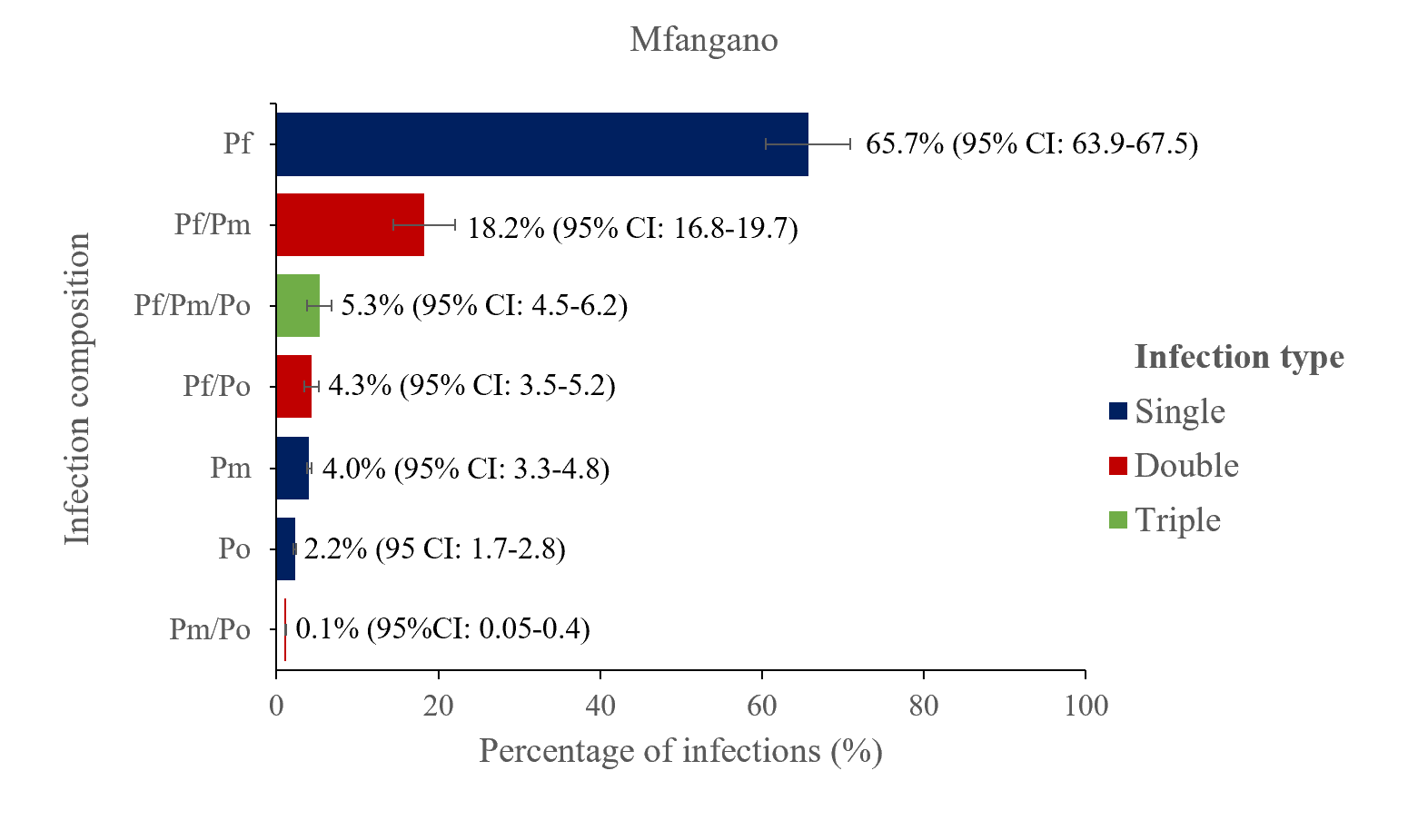
**Suplementary Figure 3.** **The proportion of *Plasmodium* mono and mixed-species infections among the positive cases by polymerase chain reaction in Mfangano island (n=2709) and Ungoye (n=2260)***.* The error bars represent a 95% confidence interval (CI). Abbreviations; CI, confidence interval; Pf, *Plasmodium falciparum;* Pm*, Plasmodium malariae;* Po*, Plasmodium ovale*.

**Supplementary Table 1.** **Changes in the relative frequency of *P. falciparum* mono- and mixed infections, as well as non-*falciparum* infections over time evaluated using multinomial logistic regression**. Adjusted relative risk ratios (RRR) of mono-infection, mixed, or non-*falciparum* mono infections each year of survey, relative to being uninfected(negative) at baseline in 2012.

| Pf mono-infection | | | | Pm mono-infection | | | Po mono-infection | | | Pf/Pm | | | Pf/Po | | | Pf/Pm/Po | | |
| --- | --- | --- | --- | --- | --- | --- | --- | --- | --- | --- | --- | --- | --- | --- | --- | --- | --- | --- |
| Year | RRR | 95% CI | P-value | RRR | 95% CI | P-value | RRR | 95% CI | P-value | RRR | 95% CI | P-value | RRR | 95% CI | P-value | RRR | 95% CI | P-value |
| 2012 | 1.00 | … | … | 1.00 | … | … | 1.00 | … | … | 1.00 | … | … | 1.00 | … | … | 1.00 | … | … |
| 2013 | 1.4 | 1.18-1.67 | <0.001 | 1.4 | 0.69-2.87 | 0.351 | 1.66 | 0.55-5.00 | 0.366 | 1.61 | 1.25-2.06 | <0.001 | 2.02 | 1.25-3.27 | 0.004 | 3.13 | 2.18-4.49 | <0.001 |
| 2014 | 0.93 | 0.80-1.08 | 0.385 | 2.12 | 1.26-3.58 | 0.005 | 2.57 | 1.10-6.00 | 0.028 | 1.34 | 1.08-1.66 | 0.007 | 1.31 | 0.83-2.06 | 0.246 | 2.01 | 1.43-2.82 | <0.001 |
| 2015 | 0.91 | 0.78-1.07 | 0.256 | 1.35 | 0.77-2.38 | 0.290 | 2.72 | 1.16-6.41 | 0.022 | 1.83 | 1.45-2.30 | <0.001 | 1.50 | 0.92-2.44 | 0.103 | 3.09 | 2.12-4.52 | <0.001 |
| 2017 | 1.07 | 0.92-1.24 | 0.364 | 2.18 | 1.31-3.64 | 0.003 | 4.58 | 2.11-9.93 | <0.001 | 2.07 | 1.69-2.54 | <0.001 | 1.68 | 1.08-2.61 | 0.020 | 2.85 | 2.03-3.99 | <0.001 |
| 2018 | 0.86 | 0.74 -1.00 | 0.056 | 1.15 | 0.65-2.05 | 0.623 | 4.04 | 1.82-8.96 | 0.001 | 1.26 | 0.99-1.60 | 0.055 | 1.55 | 0.97-2.46 | 0.065 | 1.49 | 0.97-2.27 | 0.063 |

All relative risk ratios are adjusted for age (as a categorical variable in five categories, <5, 5–10, 11–15), sex and study site. No survey was conducted in 2016.

Abbreviations: Pf*, Plasmodium falciparum;* Pm*, Plasmodium malariae;* Po*, Plasmodium ovale,* RRR; relative risk ratio. Non-falciparum mixed infection (Pm/Po) category was omitted due to consistently low frequencies observed over the years


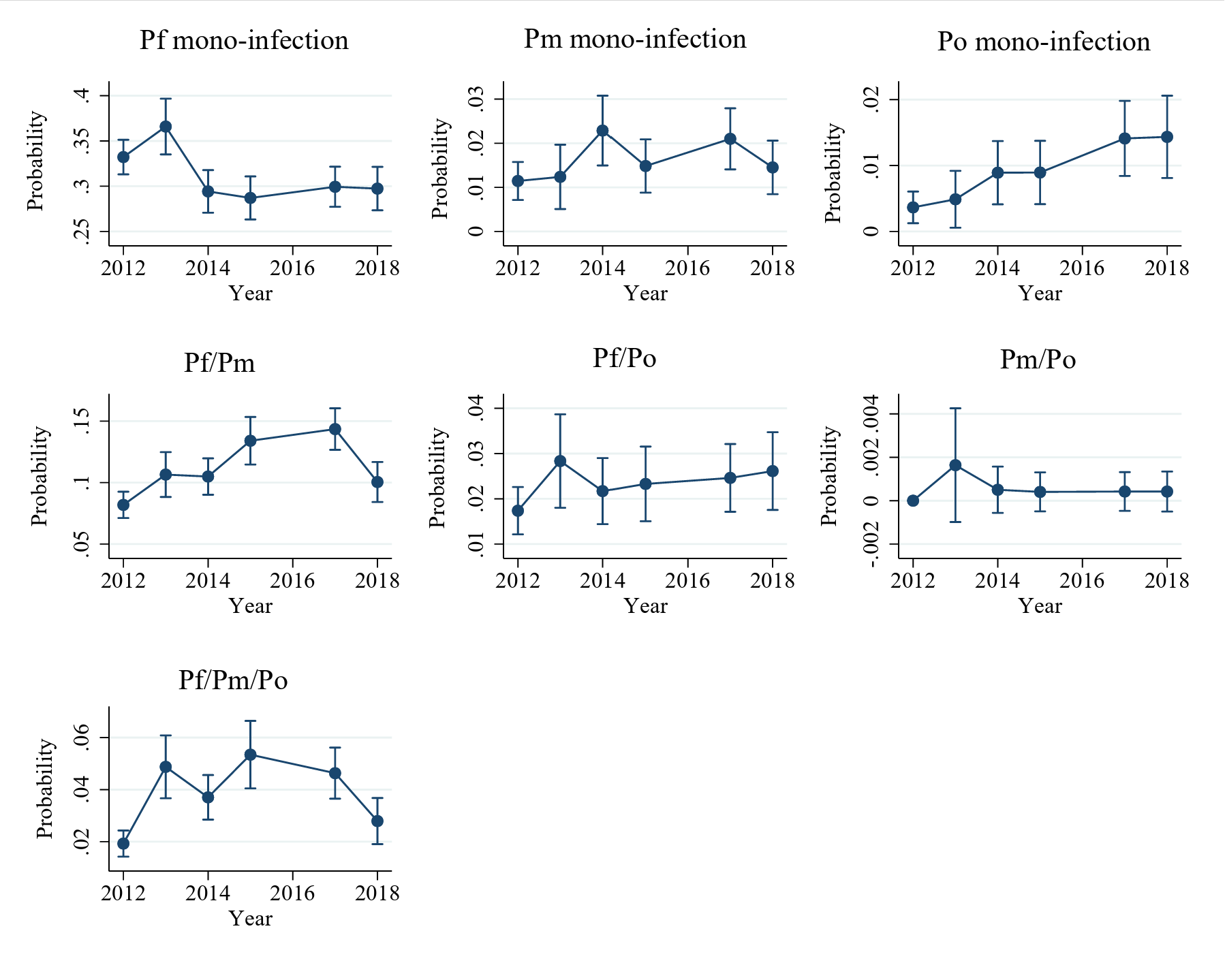


**G**

**F**

**E**

**D**

**A**

**B**

**C**

**Supplementary Figure 4. Multinomial logistic model adjusted predicted probabilities *Plasmodium* infections overtime.** A) *P. falciparum* mono-infection, B) *P. malariae* mono-infection, C) *P. ovale* mono*-*infection, D) *P. falciparum + P. malariae* mixed infection, E) *P. falciparum + P. ovale* mixed infection, F) *P. malariae + P. ovale* mixed infection, G) *P. falciparum + P. malariae* + *P. ovale* mixed infection. The model predicted the probability of infection over time. Predictions are adjusted for age, sex and study site with all covariates at their respective means. The error bars indicate the 95% confidence interval of the prediction. No surveys were conducted in 2016.
